# PhiHER2: phenotype-informed weakly supervised model for HER2 status prediction from pathological images

**DOI:** 10.1093/bioinformatics/btae236

**Published:** 2024-06-28

**Authors:** Chaoyang Yan, Jialiang Sun, Yiming Guan, Jiuxin Feng, Hong Liu, Jian Liu

**Affiliations:** College of Computer Science, Nankai University, Tianjin 300071, China; Centre for Bioinformatics and Intelligent Medicine, Nankai University, Tianjin 300071, China; College of Computer Science, Nankai University, Tianjin 300071, China; Centre for Bioinformatics and Intelligent Medicine, Nankai University, Tianjin 300071, China; College of Computer Science, Nankai University, Tianjin 300071, China; Centre for Bioinformatics and Intelligent Medicine, Nankai University, Tianjin 300071, China; College of Computer Science, Nankai University, Tianjin 300071, China; Centre for Bioinformatics and Intelligent Medicine, Nankai University, Tianjin 300071, China; The Second Surgical Department of Breast Cancer, National Clinical Research Center for Cancer, Tianjin Medical University Cancer Institute & Hospital, Tianjin 300060, China; College of Computer Science, Nankai University, Tianjin 300071, China; Centre for Bioinformatics and Intelligent Medicine, Nankai University, Tianjin 300071, China

## Abstract

**Motivation:**

Human epidermal growth factor receptor 2 (HER2) status identification enables physicians to assess the prognosis risk and determine the treatment schedule for patients. In clinical practice, pathological slides serve as the gold standard, offering morphological information on cellular structure and tumoral regions. Computational analysis of pathological images has the potential to discover morphological patterns associated with HER2 molecular targets and achieve precise status prediction. However, pathological images are typically equipped with high-resolution attributes, and HER2 expression in breast cancer (BC) images often manifests the intratumoral heterogeneity.

**Results:**

We present a phenotype-informed weakly supervised multiple instance learning architecture (PhiHER2) for the prediction of the HER2 status from pathological images of BC. Specifically, a hierarchical prototype clustering module is designed to identify representative phenotypes across whole slide images. These phenotype embeddings are then integrated into a cross-attention module, enhancing feature interaction and aggregation on instances. This yields a phenotype-based feature space that leverages the intratumoral morphological heterogeneity for HER2 status prediction. Extensive results demonstrate that PhiHER2 captures a better WSI-level representation by the typical phenotype guidance and significantly outperforms existing methods on real-world datasets. Additionally, interpretability analyses of both phenotypes and WSIs provide explicit insights into the heterogeneity of morphological patterns associated with molecular HER2 status.

**Availability and implementation:**

Our model is available at https://github.com/lyotvincent/PhiHER2

## 1 Introduction

HER2 (human epidermal growth factor receptor 2) is a crucial protein in the regulation of cell growth and division. Anomalies in HER2 expression are intricately related to the occurrence, development, and progression of various cancers, particularly breast cancer (BC) (English *et al.* 2013). Therefore, precise determination of HER2 status proves beneficial for clinicians in crafting individualized treatment plans, improving treatment efficacy, and ultimately improving patient survival rates. Immunohistochemistry (IHC) and FISH are two principal diagnostic techniques that primarily prevail in clinical settings (Wolff *et al.* 2018). Nonetheless, detecting HER2 expression remains a complex task. The diagnostic testing process requires a substantial expenditure of human resources and materials. Notably, specialized technicians are indispensable for the handling and staining of samples, causing to the overall expensive cost of testing. Furthermore, the level of HER2 expression from IHC testing is often qualitative or semi-quantitative (Chae *et al.* 2017), which introduces an element of subjectivity and uncertainty. This can potentially affect the test results and make it difficult to keep consistency among pathologists. Therefore, an efficient, accurate, and fully quantitative method is highly required for HER2 status detection in BC.

Histopathology serves as the gold standard for clinical diagnosis. The H&E pathological slides provide morphological information, accompanied by spatial organization. Nowadays, with the advent of deep learning technology and artificial intelligence, computational pathology (CPath) has experienced significant advancement by digital whole slide image (WSI) analysis (Song *et al.* 2023). It has shown remarkable progress in various tasks, including nuclei and tissue identification, tumor detection, cancer grading, subtyping, survival prediction, treatment response prediction, and prediction of molecular targets, among others (Laleh *et al.* 2022, Huang *et al.* 2023). Pathological WSI analysis is crucial, but it also offers fundamental challenges. WSIs are equipped with high-resolution and multi-scale attributes, featuring sizes that can reach up to billions of pixels. This makes it infeasible for standard CNNs to identify regions of interest (ROIs) and aggregate features. Also, it is essential to uncover morphological phenotypes and subvisual characteristics, especially in the context of tasks involving molecular-omics targets. In previous studies, Ding *et al.* (2022) proposed a graph neural network model to predict the cross-level molecular profiles of genetic mutations, copy number alterations, and functional protein expressions in colon cancer WSIs. They provided a graph structure interpretation scheme and demonstrated a wide range of molecular–histopathological associations. Lazard *et al.* (2022) introduced a deep learning approach to predict homologous recombination deficiency from BC WSIs and the phenotypic patterns relevant to genotype–phenotype relationships was analyzed. Binder *et al.* (2021) presented an explainable machine learning framework to profile molecular and clinical features from BC histology, which facilitated the assessment of the link between morphological and molecular properties. MOMA framework (Tsai *et al.* 2023) has been proposed to predict the genomics and proteomics status of cancer samples from WSIs. It identified interpretable patterns predictive of genetic profiles and multiomics aberrations. DEMoS model (Wang *et al.* 2022) was designed to predict molecular subtypes of microsatellite instability (MSI) on gastric cancer and a transformer model was developed for MSI status prediction in colorectal cancer WSIs. The model has been proved to learn morphological concepts associated with MSI-high prediction (Wagner *et al.* 2023).

Specifically, for automated prediction of HER2 status from pathological images, Farahmand *et al.* (2022) proposed an inception-v4 model on the basis of manually annotated tumor ROIs, while Hossain *et al.* (2023) developed an automated tumor ROI detection method. Both of them achieved HER2 status prediction, but they are equipped with a full supervised learning strategy, causing a tremendous computational cost on billions of patches, compared to weakly supervised learning methods. Pisula *et al.* (2023) implemented a weakly supervised multiple instance learning (MIL) method for IHC-stained tissue slides and an attention-based MIL approach ReceptorNet (Naik *et al.* 2020) was also performed for hormonal receptor and HER2 status prediction on H&E WSIs. Even, a graph neural network architecture was introduced for HER2 status prediction from WSIs of BC tissue (Lu *et al.* 2022). However, these approaches overlooked the intratumoral heterogeneity of HER2 expression (Seol *et al.* 2012) despite achieving remarkable accuracy in prediction capabilities. HER2 intratumoral heterogeneity manifests as the coexistence of positive and negative cells in tumor sections, which could potentially result in resistance to HER2-targeted therapies (Swain *et al.* 2023). This prompts us to explore how to discover morphologically representative and heterogeneous content from pathological images. Such morphological phenotypes will provide further hints for the precise prediction of HER2 status, which we refer to as phenotype-informed prediction. Recently, a vocabulary-based MIL paradigm has been introduced for the detection of lymph node metastasis and a prototype discovery module was designed to capture pathology patterns (Yu *et al.* 2023). The concept of prototype has been established to show how it can be interpreted biologically (Vu *et al.* 2023). This provides us with the potential to identify morphological phenotypes and reveal characteristics of intratumoral heterogeneity for HER2.

In this work, we present a phenotype-informed multiple instance learning architecture (PhiHER2) for automated HER2 status prediction of BC from H&E-stained WSIs. The PhiHER2 model leverages weakly supervised learning and is applicable to high-resolution WSIs, enabling the automatic identification of key regions without the requirement for manual ROI annotation. A hierarchical prototype clustering module is firstly designed to discover phenotypes that reveal the morphological patterns exhibited across pathological WSIs. Phenotype embeddings are then introduced into the MIL framework for feature aggregation with the cross-attention module. It enhances interaction among instances and captures a phenotype-based feature space equipped with intratumoral heterogeneity for HER2 status prediction. An overlapping heatmap visualization approach is developed for the interpretability analysis on WSIs. It uncovers the associations of morphological patterns with molecular HER2 status. The experimental results on two real-world datasets highlight that our proposed model effectively accounts for instance aggregation, captures WSI-level representations, and significantly improves the model performance.

## 2 Materials and methods

**Figure 1.**
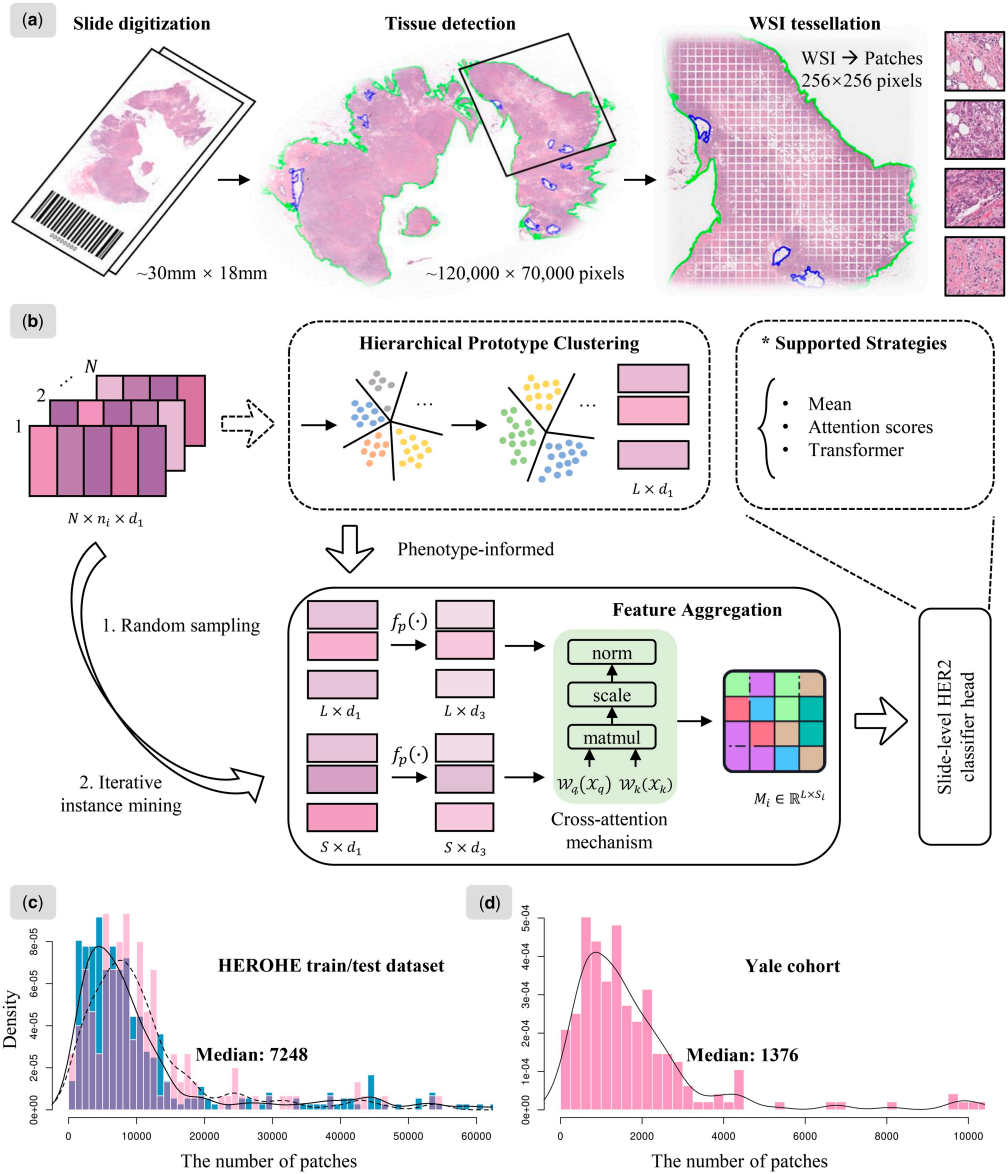
Workflow overview with (a) WSIs processing, (b) the PhiHER2 architecture, and the data distributions of the number of patches from WSIs on (c) the HEROHE dataset and (d) the Yale cohort.

Figure 1a and b illustrates the overall flowchart of our phenotype-informed framework. The workflow is based on weakly supervised learning MIL approach, including WSIs processing, feature extraction, hierarchical prototype clustering, the cross-attention module for HER2 status prediction classifier, along with the dual instance sampling strategy. In the following section, the problem will be firstly formulated, and our components will be described in detail.

### 2.1 Problem formulation

Given a WSI dataset $X={\left\{X_{i}\right\}}_{i=1}^{N}$ containing *N* slides. Each slide *X_i_* is tiled into patches ${\left\{t_{j}^{i}\right\}}_{j=1}^{n_{i}}$, where *i* and *j* denote the index of the slide and the patch, respectively. *n_i_* is the number of patches of *X_i_*. In the concept of MIL, each patch $t_{j}^{i}$ is known as an instance and the set of patches ${\left\{t_{j}^{i}\right\}}_{j=1}^{n_{i}}$ is a bag. For a classification task, there only exists known bag-level label $Y={\left\{Y_{i}\right\}}_{i=1}^{N}$ and the corresponding instance-level labels ${\left\{y_{j}^{i}\right\}}_{j=1}^{n_{i}}$ for patches are unknown. Our goal is to learn a MIL model F(·) to predict the bag-level category ${\hat{Y}}_{i}$ from this bag $X_{i}$:

(1) $${\hat{Y}}_{i}\leftarrow F(X_{i})=F({\left\{t_{j}^{i}\right\}}_{j=1}^{n_{i}})$$

F(·) can be further decomposed into three parts, the transformation function T(·) on instances, the aggregation function S(·), and the bag-level classifier head R(·)

(2) $$F(X_{i})=R(S({\left\{T(t_{j}^{i})\right\}}_{j=1}^{n_{i}}))=R(S({\left\{p_{j}^{i}\right\}}_{j=1}^{n_{i}}))=R(s^{i})$$

(3) $$p_{j}^{i}\leftarrow T(t_{j}^{i}),\;\;\;\;p_{j}^{i}\in R^{d_{1}},\;\;j=1,2,\ldots ,n_{i}$$

(4) $$s^{i}\leftarrow S({\left\{p_{j}^{i}\right\}}_{j=1}^{n_{i}}),\;\;\;\;s^{i}\in R^{d_{2}},\;\;{\left\{p_{j}^{i}\right\}}_{j=1}^{n_{i}}\in R^{n_{i}\times d_{1}}$$

T(·) function transforms each instance $t_{j}^{i}$ as a feature vector $p_{j}^{i}$ with dimensionality *d*_1_ and S(·) aggregates all instance embeddings $P_{i}={\left\{p_{j}^{i}\right\}}_{j=1}^{n_{i}}$ into a bag representation vector *s^i^*, which is used for predicting ${\hat{Y}}_{i}$ by the classifier function R(·).

Typically, T(·) and R(·) could be parameterized by neural networks optimization. The key challenge in MIL lies in how to deal with the relationships between instances in one bag in S(·) so that the model can represent efficiently and make accurate predictions. We here introduce a new function ϕ(·) into the feature aggregation function S(·) for guidance

(5) $$s^{i}\leftarrow S(\phi (\cdot ),\;{\left\{p_{j}^{i}\right\}}_{j=1}^{n_{i}})$$

(6) $${\left\{p_{k}\right\}}_{k=1}^{L}\leftarrow \phi ({\left\{P_{i}\right\}}_{i=1}^{N}),\;\;{\left\{p_{k}\right\}}_{k=1}^{L}\in R^{L\times d_{1}}$$

ϕ(·) is capable of discovering *L* phenotypes ${\left\{p_{k}\right\}}_{k=1}^{L}$ from instance embeddings among WSIs ${\left\{P_{i}\right\}}_{i=1}^{N}$.

### 2.2 Whole slide images processing

The objective of processing WSIs is to filter out background areas that are not tissue, and to tile WSIs containing billions of pixels into small patches for MIL. Here, the WSIs processing method in CLAM (Lu *et al.* 2021) is utilized for tissue detection. The non-overlapping sliding window tiling process is then executed within the detected region contours at the highest magnification, with each patch sized at 256 × 256 pixels (Fig. 1a). The number of patches obtained from WSIs could range from thousands to hundreds of thousands. Figure 1c and d illustrates the distribution of the number of patches.

### 2.3 Features extraction backbone

Feature extraction served as a transformation function T(·) is a fundamental step in MIL. A contrastive learning-based feature extraction model (Wang *et al.* 2023), pre-trained on around 15 million patches from over 32 000 WSIs, has recently emerged as a universal feature extractor within the computational pathology community. We employ it for feature extraction on the instances. This enables us to obtain 2048-dimensional vectors from tiled 256 × 256 pixel patches. The extraction of low-dimensional features helps to reduce computational complexity and facilitates the modeling of MIL on instances within a slide.

### 2.4 Hierarchical prototype clustering

The prototype clustering is the function ϕ(·) we introduce to the typical MIL framework. It is designed to minimize noise and capture representative patterns that reveal the underlying tissue heterogeneity. This helps to guide feature aggregation S(·) and obtain a WSI-level representation for the accurate prediction of HER2 status. Considering the number of instances, we develop a two-stage hierarchical prototype clustering module based on unsupervised Affinity Propagation (AP) clustering algorithm (Frey and Dueck 2007). By this way, our module can be insensitive to the initial values of the data and capable of identifying the optimal number of clusters when processing WSIs with a diverse range of instances.

The hierarchical prototype clustering module is a stacked structure with multiple AP cluster classes. Abstractly, taking the ${\left\{P_{i}\right\}}_{i=1}^{N}$ as input, the first-stage AP cluster $M_{i}^{ap}$ is iteratively conducted on each bag with instance features *P_i_*, ultimately yielding the cluster centers ${\left\{p_{m}^{i}\right\}}_{m=1}^{c_{i}}$, where *m* is the index

(7) $$\begin{array}{cc} & {\left\{p_{m}^{i}\right\}}_{m=1}^{c_{i}}\leftarrow M_{i}^{ap}(P_{i}),\;\;\;\;i=1,2,\ldots ,N\\ & {\left\{p_{m}^{i}\right\}}_{m=1}^{c_{i}}\subseteq P_{i},\;\;\;{\left\{p_{m}^{i}\right\}}_{m=1}^{c_{i}}\in R^{c_{i}\times d_{1}},\;c_{i}\in N,\;c_{i}\ll n_{i} \end{array}$$

${\left\{p_{m}^{i}\right\}}_{m=1}^{c_{i}}$ is a subset of instance features *P_i_*. *c_i_* represents the number of cluster centers derived from the bag features *P_i_*, and *c_i_* is significantly smaller than the number of instances *n_i_*. Subsequently, the second-stage AP cluster $M_{a}^{ap}$ is applied to cluster centers *C^N^* across WSIs

(8) $$C^{N}={\left\{p_{m}^{1}\right\}}_{m=1}^{c_{1}}\cup {\left\{p_{m}^{2}\right\}}_{m=1}^{c_{2}}\cup \cdots \cup {\left\{p_{m}^{N}\right\}}_{m=1}^{c_{N}}$$

(9) $${\left\{p_{k}\right\}}_{k=1}^{L}\leftarrow M_{a}^{ap}(C^{N}),\;\;{\left\{p_{k}\right\}}_{k=1}^{L}\subseteq C^{N},L\in N,L<\sum_{i=1}^{N}c_{i}$$

*C^N^* is the union of ${\left\{p_{m}^{i}\right\}}_{m=1}^{c_{i}}$ from the first-stage AP cluster, and ${\left\{p_{k}\right\}}_{k=1}^{L}\in R^{L\times d_{1}}$ denotes the final centers after clustering on *C^N^*. In this way, a collection of *L* unique phenotypes is discovered from WSIs, revealing diverse morphological patterns.

In the implementation of the AP cluster algorithm, we employ the power operation of the negative normalized Euclidean distance (Yu *et al.* 2023) to measure the similarity between the data points in both stages. We set the damping factor to 0.5 to avoid numerical oscillations during the iteration process, as mentioned in Frey and Dueck (2007). It is noteworthy that the hierarchical prototype clustering module is performed on the training data only. When inferring on the test data points, the pre-clustered prototype representation is loaded and utilized for guidance.

### 2.5 Feature aggregation with cross-attention

Cross-attention is an attention mechanism within the transformer architecture (Vaswani *et al.* 2017) that integrates two distinct embedding sequences. To learn a better understanding of tissue heterogeneity with phenotype embeddings, we adapt the cross-attention module for the feature aggregation S(·) in our MIL.

Specifically, for the input instance features $P_{i}\in R^{n_{i}\times d_{1}}$ of a WSI and phenotype embeddings ${\left\{p_{k}\right\}}_{k=1}^{L}\in R^{L\times d_{1}}$, a weight-shared fully connected projection layer $f_{p}(\cdot )$ is firstly utilized to reduce dimensionality, obtaining ${\hat{P}}_{i}$ and ${\left\{{\hat{p}}_{k}\right\}}_{k=1}^{L}$, respectively

(10) $$\begin{array}{cc} {\hat{P}}_{i}= & f_{p}(P_{i}),\;\;\;\;{\left\{{\hat{p}}_{k}\right\}}_{k=1}^{L}=f_{p}({\left\{p_{k}\right\}}_{k=1}^{L})\\ & f_{p}(\cdot ):R^{d_{1}}\to R^{d_{3}} \end{array}$$

Then, they are sent to the cross-attention part for interaction. The cross-attention module includes inputs with Key and Query, where the phenotype embeddings ${\left\{{\hat{p}}_{k}\right\}}_{k=1}^{L}$ are treated as Query, and instance features ${\hat{P}}_{i}$ are represented as Key. Query and Key pass through respective linear transformation layers $w_{q}(\cdot )$ and $w_{k}(\cdot )$ to obtain the *Q* and *K_i_* matrices. At this point, the product of the two matrices yields a new measurement matrix *M_i_*, followed by a normalized operation

(11) $$M_{i}=\frac{QK_{i}^{T}}{\left(||Q|{|}_{2}\cdot ||K_{i}|{|}_{2}\right)},\;\;\;\;M_{i}\in R^{L\times n_{i}}$$

(12) $$Q=w_{q}({\left\{{\hat{p}}_{k}\right\}}_{k=1}^{L}),\;K_{i}=w_{k}({\hat{P}}_{i})$$

Here, $||\cdot |{|}_{2}$ represents the L2 norm and matrix *K_i_* is transposed when computing. In the standard transformer architecture, this measurement matrix serves as the attention coefficients, which are utilized to compute a new representation by multiplying with the Key. In our approach, guided by the phenotype embeddings ${\left\{p_{k}\right\}}_{k=1}^{L}$, each column of the measurement matrix ${\left\{M_{j}^{i}\right\}}_{j=1}^{n_{i}}$ has been projected into a new feature space $R^{L}$ corresponding to a specific set of embeddings *Q*. Consequently, we take this transformed matrix *M_i_* as the bag representation *s^i^* for the image *X_i_*. This facilitates interaction and integration between phenotype embeddings ${\left\{p_{k}\right\}}_{k=1}^{L}$ with instance features *P_i_* from each WSI.

### 2.6 HER2 status classifier head and loss optimization

On the basis of the phenotype-based bag-level representation *s^i^*, different classifier heads R(·) can be applied to matrix *M_i_* for HER2 status prediction. We explore classifiers including mean-based, attention score-based, and transformer-based heads.

- *The mean-based linear head* can be described as an average on the representation matrix *M_i_* along the axis of instances, resulting in a vector $\bar{M_{i}}\in R^{L}$. This vector is passed into the MLP layer for predicting ${\hat{Y}}_{i}$. The MLP layer consists of a linear layer, a ReLU activation function and a linear output layer followed by a softmax function.
- *The attention score-based fusion classifier* is denoted as follows: learning instance-level attention scores on the matrix *M_i_*, operating a weighted sum on them (Ilse *et al.* 2018), and predicting ${\hat{Y}}_{i}$ by the MLP layer.
- *The transformer classifier* consists of a transformer encoder (Vaswani *et al.* 2017) with an optimized class token and the multi-head self-attention mechanism. It uses a fully connected layer to predict ${\hat{Y}}_{i}$ based on the class token.

We employ cross-entropy loss as the overall objective function L(·) to optimize our PhiHER2 model

(13) $$\begin{array}{cc} L(\cdot ) & =-\frac{1}{B}\sum_{i}^{B}\left(Y_{i}\;\text{log}({\hat{Y}}_{i})+(1-Y_{i})\log(1-{\hat{Y}}_{i})\right)\\ & \left\{{\hat{\theta }}_{S},{\hat{\theta }}_{R}\}\leftarrow \arg\min_{\left\{\theta_{S},\theta_{R}\right\}}L(\cdot \right) \end{array}$$

where *B* is the batch size, *Y_i_* denotes the slide-level label of HER2 status, and ${\hat{Y}}_{i}$ represents the predicted probability for this WSI. $\theta_{S}$ and $\theta_{R}$ are the parameters in S(·) and R(·), and argmin(·) aims to find the parameters that minimize the function L(·).

### 2.7 Dual instance sampling

In our work, we introduce a dual instance sampling strategy designed to function as a bag-level data augmentation technique in MIL. This strategy is composed of two components: random sampling and iterative instance mining.

Initially, for a given bag *X_i_* in the dataset, we randomly sample a subset with *R* instance embeddings ${\left\{p_{j}^{i}\right\}}_{j=1}^{R}\subseteq {\left\{p_{j}^{i}\right\}}_{j=1}^{n_{i}}$ to be included in each training epoch process. Subsequently, we employ a score-based instance selector to mine *S* instance embeddings ${\left\{p_{j}^{i}\right\}}_{j=1}^{S}\subseteq {\left\{p_{j}^{i}\right\}}_{j=1}^{R}$ with high scores during the iteratively training process. Here, *R* and *S* are pre-defined numbers. The score-based instance selector utilizes a linear layer to compute vector scores for iterative mining, and it is positioned prior to the weight-shared fully connected projection layer $f_{p}(\cdot )$ (defined in Section 2.5). The dual instance sampling strategy is engineered to be a plug-and-play component, ensuring the simplicity of our PhiHER2 architecture is maintained.

## 3 Experiments and results

### 3.1 Data description

The HEROHE dataset was from the HEROHE challenge, which aimed to predict HER2 status directly from H&E-stained pathological images (Conde-Sousa *et al.* 2022). It includes a training dataset and an independent test dataset. The training dataset consists of 360 WSIs of invasive BC tissue samples, and the test dataset contains 150 WSIs. The corresponding slide-level ground truth labels (positive or negative) were derived from IHC and ISH tests according to clinical practice guidelines (Wolff *et al.* 2018). All slides in both training and test datasets were scanned at 20× magnification with 0.243 μm per pixel and originated from different patients. No tumor region annotations or IHC slides were provided.

The Yale HER2 cohort was also collected (Farahmand *et al.* 2022). It contains 191 HER2 positive and negative invasive BC samples, including 93 positive and 98 negative slides. The H&E-stained WSIs generated at Yale School of Medicine were scanned by Aperio ScanScope at 20× magnification with 0.497 μm per pixel. They were annotated with ROIs associated to tumor areas on invasive carcinoma by a senior breast pathologist and the ROI annotation files were included in the dataset. Please keep in mind that the ROIs mentioned here were not used in our training process. Instead, we took them for comparative analysis with slide-level heatmap visualization to interpret the results.

### 3.2 Experimental designs

Implementation details and evaluation criteria can be found in the supplementary materials (Supplementary Sections S1 and S2).

### 3.3 Comparison with state-of-the-art methods

**Figure 2.**
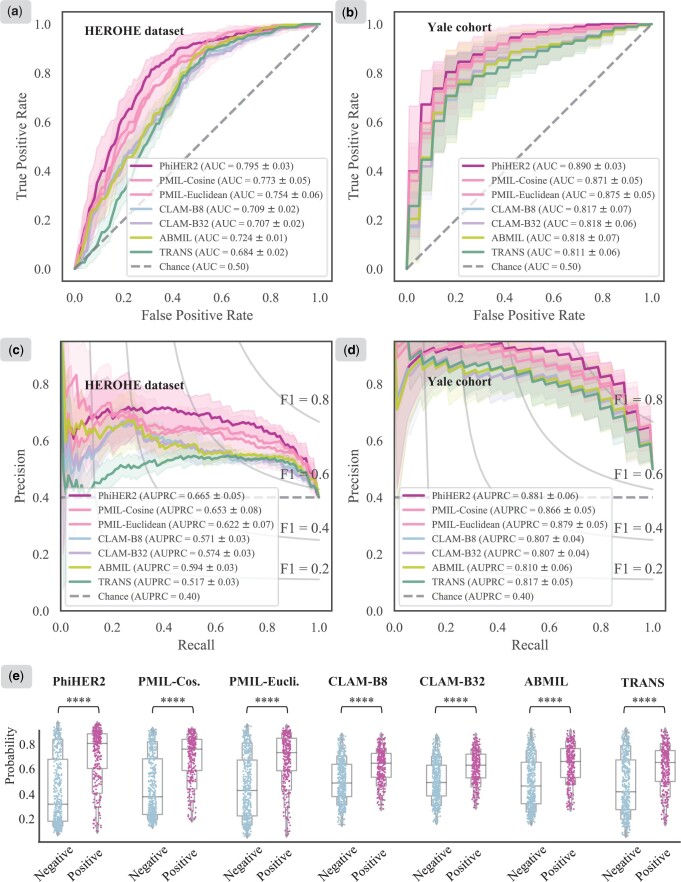
Evaluation performance and comparative analysis for HER2 status prediction. (a, b) ROCs for PhiHER2 and comparative models tested on the HEROHE and Yale cohort. (c, d) PR curves for our PhiHER2 and comparative models evaluated on both datasets. (e) The probability distribution of predictions on different HER2 status groups from the HEROHE dataset.

To verify the effectiveness of our PhiHER2 model, we chose several state-of-the-art weakly supervised methods (ABMIL (Ilse *et al.* 2018), CLAM (Lu *et al.* 2021), PMIL (Yu *et al.* 2023), and TRANS (Wagner *et al.* 2023)) for comparison. Brief introductions of these methods are listed in Supplementary Section S3. We conducted a systematic evaluation on the performance of our PhiHER2 against comparative methods. The quantitative results on positive F1-score (posF1), weighted averaged Precision (wPRC), Recall (wREC), F1-score (wF1), and balanced accuracy (bACC) are shown in Table 1. The evaluation analysis involved ROC and PR curves, along with average AUC and AUPRC values, are illustrated in Fig. 2a–d. The comparison of inference time cost is reported in Supplementary Table S1, and the performance of our model across 5-time experiments is depicted in Supplementary Fig. S2.

**Table 1.** Performance comparison of PhiHER2 and comparative methods on the HEROHE and Yale set.

Dataset	Method	posF1	wPRC	wREC	wF1	bACC
HEROHE	**PhiHER2**	**0.706 ± 0.02**	**0.762 ± 0.03**	**0.721 ± 0.02**	**0.722 ± 0.02**	**0.741 ± 0.02**
	PMIL-Cosine	0.693 ± 0.04	0.750 ± 0.05	0.700 ± 0.05	0.700 ± 0.05	0.724 ± 0.04
	PMIL-Euclidean	0.682 ± 0.06	0.738 ± 0.06	0.691 ± 0.05	0.692 ± 0.05	0.714 ± 0.06
	CLAM-B8	0.645 ± 0.02	0.701 ± 0.02	0.643 ± 0.02	0.642 ± 0.02	0.671 ± 0.02
	CLAM-B32	0.636 ± 0.01	0.691 ± 0.02	0.635 ± 0.01	0.634 ± 0.01	0.662 ± 0.01
	ABMIL	0.651 ± 0.01	0.706 ± 0.02	0.655 ± 0.01	0.655 ± 0.01	0.680 ± 0.01
	TRANS	0.612 ± 0.08	0.678 ± 0.05	0.635 ± 0.03	0.634 ± 0.03	0.652 ± 0.05
Yale	**PhiHER2**	**0.820 ± 0.05**	**0.829 ± 0.05**	**0.819 ± 0.05**	**0.817 ± 0.05**	**0.819 ± 0.05**
	PMIL-Cosine	0.794 ± 0.08	0.799 ± 0.07	0.796 ± 0.07	0.795 ± 0.08	0.796 ± 0.07
	PMIL-Euclidean	0.806 ± 0.07	0.807 ± 0.07	0.803 ± 0.08	0.802 ± 0.08	0.803 ± 0.08
	CLAM-B8	0.748 ± 0.09	0.773 ± 0.08	0.764 ± 0.08	0.762 ± 0.08	0.764 ± 0.08
	CLAM-B32	0.756 ± 0.08	0.778 ± 0.08	0.771 ± 0.08	0.769 ± 0.08	0.771 ± 0.08
	ABMIL	0.754 ± 0.08	0.775 ± 0.08	0.767 ± 0.08	0.766 ± 0.08	0.767 ± 0.08
	TRANS	0.751 ± 0.09	0.768 ± 0.09	0.760 ± 0.09	0.758 ± 0.09	0.760 ± 0.09

The results were evaluated and averaged across 5-time experiments. Mean and standard deviation are reported, and the best results are indicated in **bold**.

The results demonstrate superior performance of our PhiHER2 in accurately identifying HER2 status from BC WSIs. It achieved the best AUC values of 0.795 and 0.890 on the HEROHE dataset and the Yale cohort, respectively (Fig. 2a–b). Moreover, the average AUPRC value of 0.881 (Fig. 2d) shows that our model achieved the best recall with precision on the Yale cohort. Due to the strong class imbalance ratio of 1.5 in the HEROHE dataset, models’ AUPRCs were relatively inferior compared to the AUCs. Nevertheless, our proposed PhiHER2 still attained the highest AUPRC value of 0.665 (Fig. 2c). The baseline ABMIL and the CLAM method, enhanced with instance attention scores, yielded acceptable but unremarkable results for the specific molecular HER2 status prediction task on HEROHE and Yale WSIs (Table 1, Fig. 2a–d). The TRANS method underperformed other comparative methods. We attribute this observation to the data volume, considering their collection of over 20 000 H&E slides (Wagner *et al.* 2023). Moreover, the TRANS model, functioning as a general transformer-based pipeline for end-to-end biomarker prediction, falls short in accounting for the heterogeneity of morphological patterns. The PMIL-Cosine and PMIL-Euclidean methods outperformed CLAM and ABMIL on both datasets. This points out the advantages of phenotype-informed architecture. We further categorized the comparative models into two groups (phenotype-guided versus non-phenotype-guided) and reported the evaluation performance (Supplementary Fig. S3). We found that the first group consistently provides better performance, confirming the superiority of the concept of phenotype guidance. It is noteworthy that PhiHER2 displayed lower variance than the PMIL (Table 1). This indicates that our model has a capability in dealing with data bias and excels in accuracy and robustness when predicting HER2 status from WSIs.

Given that the HEROHE dataset was from a competition (Conde-Sousa *et al.* 2022), we benchmarked our results against the top entries on the public leaderboard (Supplementary Table S2). Our method outperformed the first-place entry, achieving a 2.6% improvement (0.706 versus 0.68). In terms of the AUC metric, our method obtained a notable improvement of 8.5% (0.795 versus 0.71) over the first-place entry. For the Yale cohort, we compared with the results presented in Farahmand *et al.* (2022). Our model significantly outperformed their unannotated two-way classifier (AUC values: 0.890 versus 0.82). In particular, our model operating without any tumor ROIs annotation, achieved or even exceeded the performance of their tumor-annotation-based models, with AUC value of 0.890 compared to 0.89 for their annotated two-way classifier and 0.88 for the annotated three-way classifier. This implicitly suggests that our model can identify key tumor regions. We will provide a detailed interpretability analysis of these findings in Section 3.7.

#### 3.3.1 Qualitative evaluation

We investigated the distribution of predictions on different HER2 status groups. The predicted probabilities for HER2-positive on the HEROHE test cases were examined (Fig. 2e). It could be observed that our method exhibits the widest gap between the median values of the prediction probabilities across different HER2 status groups. Our model is capable of a better alignment between the prediction and the true HER2 status, with cases of the negative being more frequently predicted towards the lower end of the scale, and those of the positive being more often predicted towards the upper end. These findings suggest that our PhiHER2 has the most significant discrimination between HER2-positive and negative cases, highlighting the effectiveness of our method.

### 3.4 Ablation study

To validate the effectiveness of our proposed hierarchical prototype clustering structure and the cross-attention module, we adopted four distinct experimental configurations for comparison.

(i) Cluster-PT: The model with a cross-attention module and phenotype embeddings derived from hierarchical prototype clustering structure to provide guidance. (ii) Rand-PT: Fixed random vectors served as phenotype embeddings in the cross-attention module for direct inference. Random values were sampled from a uniform distribution, with dimensions and quantity identical to those of the cluster phenotype embeddings. (iii) Initial-PT: Random vectors initialized with a uniform distribution served as phenotype embeddings. These vectors could be iteratively optimized during the training process. (iv) Non-PT: The model with a cross-attention module but no phenotype guidance. In this configuration, the cross-attention module reverts to a self-attention mechanism. We also included the ABMIL method as baseline for comparison.

**Figure 3.**
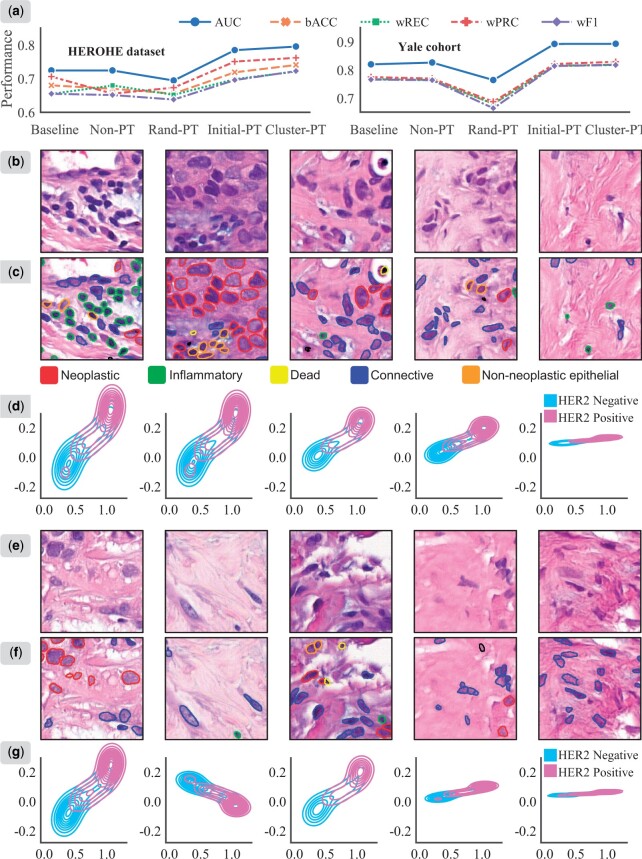
Visualization of (a) ablation results for cluster phenotype guidance with the cross-attention module evaluated on HEROHE and Yale dataset, (b, e) representative phenotypes derived from the hierarchical prototype clustering module on the HEROHE dataset, (c, f) cell segmentation and classification results overlaid on phenotype patches, where different colors mark different cell types, and (d, g) 2D density plots showing the distribution of phenotype embeddings and model prediction scores for different HER2 status groups, where the *x*-axis denotes the model prediction scores and the *y*-axis represents the specific feature values.

The architectures of experimental configurations and the comparative models for the following subsections are further illustrated in Supplementary Fig. S4. The results for these models are reported in Fig. 3a and Supplementary Table S3.

#### 3.4.1 Effectiveness of phenotype guidance

We can clearly see that Cluster-PT consistently outperformed the baseline ABMIL and Non-PT on both the HEROHE set and the Yale cohort. Cluster-PT obtained an average AUC value of 0.893, marking a significant improvement of 6.7% over Non-PT and 7.3% over the baseline on the Yale cohort (Supplementary Table S3). A comparable enhancement in performance is also evident in the results on the HEROHE dataset. Taken together, these results reinforce that phenotype guidance in Cluster-PT is effective in exploring relations between representative embeddings and the instance features of WSIs, thereby aggregating distinguishing representations.

#### 3.4.2 Effectiveness of hierarchical prototype clustering

Cluster-PT approach achieved superior performance compared to both Rand-PT and Initial-PT, with respective AUC improvements of 10.1% and 1.0% on the HEROHE dataset (Supplementary Table S3). It suggests that phenotypes can be enhanced through the incorporation of our designed hierarchical prototype clustering. The tissue phenotypes corresponding to the prototype embeddings derived from the hierarchical prototype clustering module on both datasets is provided (Fig. 3b and e, Supplementary Fig. S5). We found that the clustering module captures typical phenotypes within WSIs. Expert pathologists characterized these regional tissue phenotypes as dense tumor cellularity, a discrete distribution of tumor cells, abundant stromal fibrosis, the presence of tumor-infiltrating lymphocytes, and white background.

We employed pre-trained Hover-Net (Graham *et al.* 2019) for cell segmentation and classification on those regional tissue phenotypes. This enabled us to obtain the cellular spatial composition of five cell types, including neoplastic, inflammatory, connective, dead, and non-neoplastic epithelial cells (Fig. 3c and f, Supplementary Fig. S6). It could be observed that these phenotypes depict differences in spatial cellular composition. For example, certain phenotype patches exhibit a high abundance of locally-aggregated inflammatory cells and a higher percentage of tumor (neoplastic) cells, while others show loosely distributed stromal (connective) cells interconnected with tumor cells. These observations align with those made by expert pathologists. The representative phenotypes significantly reveal distinct cellular spatial compositions and morphological patterns in pathological images. It potentially promotes explainable insights into the spatial morphological heterogeneity of BC.

We also conducted a further analysis on the prototype embeddings with model prediction scores to compare HER2-positive (denoted in purple) with HER2-negative (blue) cases. A distinct separation is evident between the two groups according to the 2D density plots (Fig. 3d and g). The observations clearly indicate an association between pathologically morphological patterns and molecular HER2 status.

#### 3.4.3 Effectiveness of the cross-attention module

**Figure 4.**
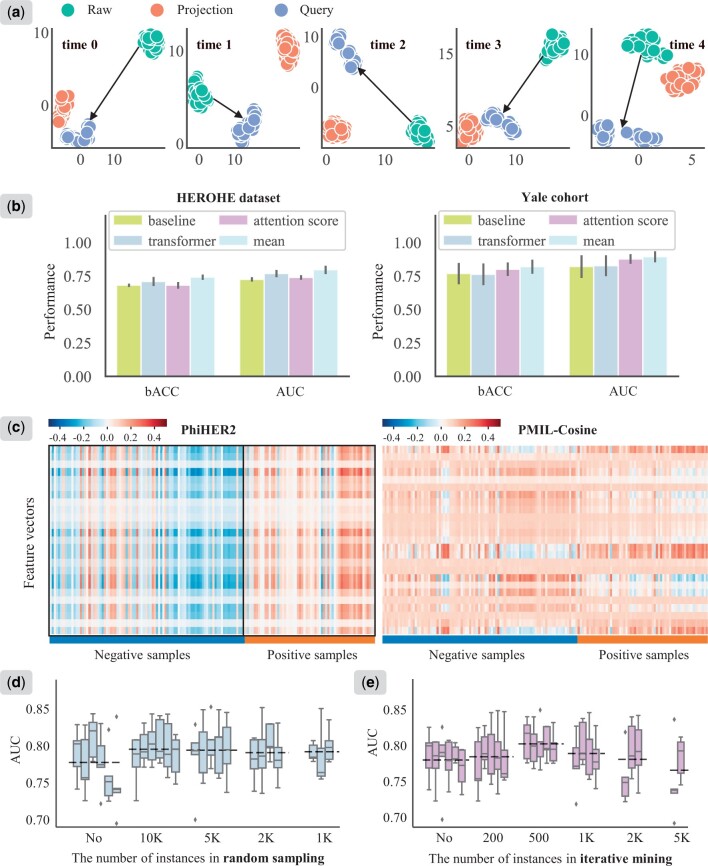
Visualization of (a) phenotype embeddings for 5-time models on the HEROHE dataset, (b) evaluation performance with different classifier head strategies on the HEROHE dataset and the Yale cohort, (c) WSI-level representation heatmaps for PhiHER2 and PMIL methods, where each row represents a specific dimension in the WSI-level feature vector, each column represents a sample with negative (blue) or positive (orange) HER2 status and the color in the heatmap indicates the value intensity of the feature vectors. (d–e) Illustration of data efficiency for dual instance sampling regarding the number of instances in (d) random sampling and (e) iterative mining on the HEROHE dataset.

Initial-PT demonstrated a clear advantage over Rand-PT, with AUC values of 0.785 on the HEROHE dataset and 0.892 on the Yale cohort (Supplementary Table S3). However, both Rand-PT and Initial-PT models were initialized randomly. This prompts us to investigate the degree to which prototype embeddings are affected by model optimization within the cross-attention module. We extracted the transformation embeddings of phenotypes across various linear layers (input, projection, and query) within the cross-attention module. These embeddings were denoted as Raw, Projection, and Query, respectively. The uniform manifold approximation and projection was applied to the embeddings for dimensionality reduction, allowing visualization of phenotypes in a two-dimensional space (Fig. 4a). We found that the prototype embeddings from the same layer tend to cluster together, whereas the representations from different layers are well separated. The arrows roughly mark the distances between Raw and Query embeddings. This implies that the iteratively optimized cross-attention module guiding by prototypes can further facilitate the transformation of phenotypes into an alternative feature space. It explains why Initial-PT outperforms Rand-PT and validates the critical role of the cross-attention module in our PhiHER2 model.

### 3.5 Robustness of classifier head strategies

In this experiment, we compared the performance of our PhiHER2 model when coupled with different classifier heads, covering those based on mean operation, attention scores, and transformer strategies (Fig. 4b, Supplementary Table S4). We also reported the results of the baseline ABMIL model. The performance indicates that our PhiHER2 remains relatively robust with respect to different classifier head strategies and these models outperform the baseline ABMIL method across both the HEROHE dataset and the Yale cohort. It highlights that our phenotype-informed framework is insensitive to the classifier heads. Notably, the model equipped with the mean-based classifier head yielded the best performance compared to others. This appears to suggest that the PhiHER2 model has better consistency among instance embeddings within a single WSI. We thus turn to obtain the WSI-level representation vector before the classifier head and visualize them by embedding heatmaps (Fig. 4c, Supplementary Fig. S7). Our phenotype-informed aggregated features reveal a significant distinction between the HER2-positive and negative groups (Fig. 4c). This is in line with the observations from Fig. 3d and g. Additionally, PhiHER2 displays greater embedding consistency than those of PMIL and non-phenotype-guided methods (ABMIL and CLAM). This results in other methods showing a greater dependency on the classifier head, whereas our PhiHER2 can facilitate feature aggregation and enhance performance through a concise mean-based classifier head strategy.

### 3.6 Efficiency with dual instance sampling

To assess the efficiency of the dual instance sampling strategy for our PhiHER2, we conducted experiments by varying the number of instances in random sampling and iterative mining. We established these sampling values by scaling the median value of the dataset accordingly (see Fig. 1c–d). For the HEROHE dataset, with a median value of 7248, we chose fixed sampling values of 10 000, 5000, 2000, 1000, 500, and 200 for comparison. For the Yale cohort, which has a smaller median value of 1376, we selected 2000, 1000, 500, 200, and 100. Besides, we performed experiments with no instance sampling to ensure a fair comparison.

The results are presented in Fig. 4d and e and Supplementary Fig. S8. We can observe the following empirical facts. The random instance sampling strategy brings performance improvements compared to the model with no instance sampling, and the latter also exhibits significant performance fluctuations. The model performance remains generally consistent when the number of instances in random sampling decreases. This shows that random instance sampling acting as a data augmentation in MIL enhances the training efficiency and performance of the model (Naik *et al.* 2020, Cao *et al.* 2023). The model with 500 instances of iterative mining achieved optimal performance, with a 2.3% improvement over the model with no iterative mining on the HEROHE dataset (Fig. 4e). Similar findings are observed from the results on the Yale cohort (Supplementary Fig. S8). These results underscore the contribution of our proposed dual instance sampling strategy.

### 3.7 WSIs interpretability analysis

For weakly supervised deep learning models in medical applications, the interpretability is vital and decisions should be visually explainable for clinicians. To this end, we implemented an overlapping heatmap visualization method to determine the importance of the region and interpret the morphological patterns associated with the molecular status of HER2. Specifically, for one WSI, the PhiHER2 model processed and generated instance-level predictions without employing the mean operation within its classifier head. The model’s predicted class probabilities for each instance were obtained and normalized to fall within the interval of 0 and 1. These normalized scores of instances were then mapped to their corresponding spatial locations within the WSI and visualized as a heatmap, which effectively highlights the regions where the model exhibits the highest confidence.

**Figure 5.**
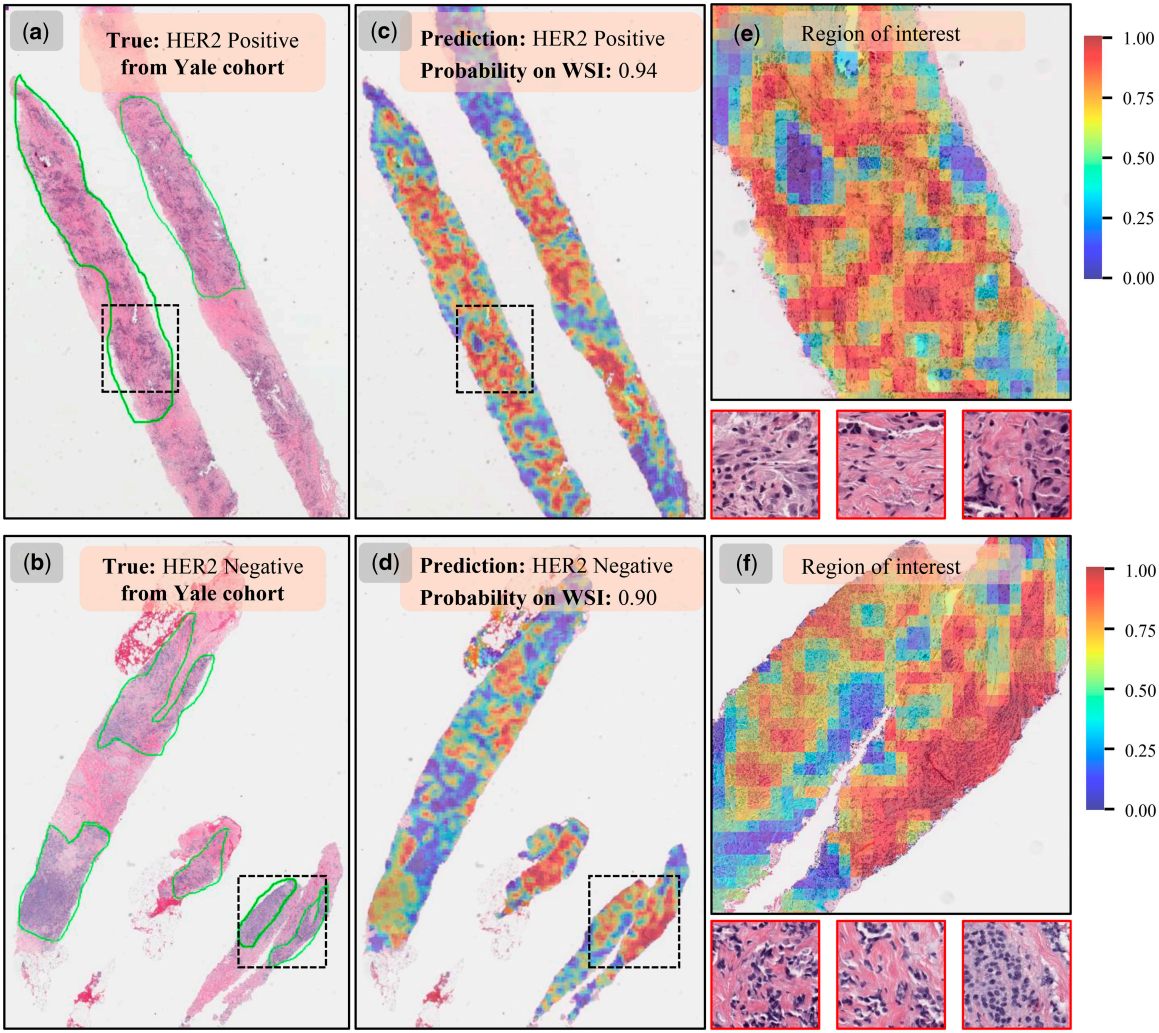
WSI-level overlapping heatmap visualization for model interpretability. (a, b) Two pathological images with manual ROI annotations derived from the Yale cohort. (c, d) The corresponding attention heatmaps overlapped on raw WSIs. Large values (red) means a high contribution to the model’s prediction, small values (blue) a low contribution. (e, f) Selected ROIs for zooming in to observe detailed tissue regions. Three tissue patches with the highest probabilities are also presented.

Fine-grained heatmaps overlapped on WSIs from the Yale cohort and the HEROHE testset are visualized (Fig. 5a–d, Supplementary Fig. S9). One can find that the WSI heatmaps exhibit a high degree of consistency with the expert annotations of ROIs in HER2-positive and negative cases (Fig. 5c–d). Our trained weakly supervised PhiHER2 model is able to identify and weight ROIs related to HER2 status using slide-level labels only. We can observe the distribution of attention heatmaps on distinct tissue ROIs (Fig. 5e and f). Most of the high-contribution areas are located in tumor tissue. The representative patches with the highest probabilities mostly derive from tumor areas. This reveals the interpretability of which tissue patterns are more meaningful for WSI-level HER2 status prediction decision. It has the potential to provide clinicians deep insights into morphological patterns of region heterogeneity of HER2 expression.

## 4 Conclusion

In this work, we introduce PhiHER2, a phenotype-informed weakly supervised learning architecture designed for the prediction of HER2 status from BC WSIs. PhiHER2 leverages heterogeneous prototype embeddings to guide feature aggregation in the MIL framework. The proposed hierarchical prototype clustering module discovers typical phenotypes, which account for the tumor heterogeneity exhibited in WSIs. Additionally, the cross-attention module enhances instance interactions and captures a feature space based on the representative phenotypes. The concept of phenotype guidance in the cross-attention mechanism proves to be flexible and robust across various classifier head strategies. The experiments demonstrate that the PhiHER2 model achieved a remarkable improvement in performance over existing methods on both the HEROHE dataset and the Yale cohort. Interpretative analysis of phenotype instances and WSI heatmaps offers valuable insights into the heterogeneity of tumor morphological patterns associated with the molecular status of HER2. Our model exhibits the potential to serve as a reliable framework for other studies, and its capability of handling more clustering algorithms and cross-dataset applications will be investigated in our future work.

## Supplementary Material

btae236_Supplementary_Data

## Data Availability

All the data used in this manuscript are publicly available. The HEROHE dataset derived from the ECDP2020 challenge can be obtained from the public domain: https://ecdp2020.grand-challenge.org/Dataset/. The Yale HER2 cohort is available in The Cancer Imaging Archive, at https://doi.org/10.7937/E65C-AM96.

## References

[btae236-B1] Binder A , Bockmayr M, Hägele M et al Morphological and molecular breast cancer profiling through explainable machine learning. Nat Mach Intell 2021;3:355–66.

[btae236-B2] Cao L , Wang J, Zhang Y et al E2efp-mil: end-to-end and high-generalizability weakly supervised deep convolutional network for lung cancer classification from whole slide image. Med Image Anal 2023;88:102837. 37216736 10.1016/j.media.2023.102837

[btae236-B3] Chae YK , Arya A, Chiec L et al Challenges and future of biomarker tests in the era of precision oncology: can we rely on immunohistochemistry (IHC) or fluorescence in situ hybridization (FISH) to select the optimal patients for matched therapy? Oncotarget 2017;8:100863–98. 29246028 10.18632/oncotarget.19809 PMC5725070

[btae236-B4] Conde-Sousa E , Vale J, Feng M et al Herohe challenge: predicting Her2 status in breast cancer from hematoxylin–eosin whole-slide imaging. J Imaging 2022;8:213. 36005456 10.3390/jimaging8080213 PMC9410129

[btae236-B5] Ding K , Zhou M, Wang H et al Spatially aware graph neural networks and cross-level molecular profile prediction in colon cancer histopathology: a retrospective multi-cohort study. Lancet Digit Health 2022;4:e787–95. 36307192 10.1016/S2589-7500(22)00168-6

[btae236-B6] English DP , Roque DM, Santin AD. Her2 expression beyond breast cancer: therapeutic implications for gynecologic malignancies. Mol Diagn Ther 2013;17:85–99. 23529353 10.1007/s40291-013-0024-9 PMC3660991

[btae236-B7] Farahmand S , Fernandez AI, Ahmed FS et al Deep learning trained on hematoxylin and eosin tumor region of interest predicts Her2 status and trastuzumab treatment response in Her2+ breast cancer. Mod Pathol 2022;35:44–51. 34493825 10.1038/s41379-021-00911-w PMC10221954

[btae236-B8] Frey BJ , Dueck D. Clustering by passing messages between data points. Science 2007;315:972–6. 17218491 10.1126/science.1136800

[btae236-B9] Graham S , Vu QD, Raza SEA et al Hover-net: simultaneous segmentation and classification of nuclei in multi-tissue histology images. Med Image Anal 2019;58:101563. 31561183 10.1016/j.media.2019.101563

[btae236-B10] Hossain MS , Shahriar GM, Syeed MM et al Region of interest (ROI) selection using vision transformer for automatic analysis using whole slide images. Sci Rep 2023;13:11314. 37443188 10.1038/s41598-023-38109-6 PMC10344922

[btae236-B11] Huang Z , Shao W, Han Z et al Artificial intelligence reveals features associated with breast cancer neoadjuvant chemotherapy responses from multi-stain histopathologic images. NPJ Precis Oncol 2023;7:14. 36707660 10.1038/s41698-023-00352-5 PMC9883475

[btae236-B12] Ilse M , Tomczak J, Welling M. Attention-based deep multiple instance learning. In *International Conference on Machine Learning*, *PMLR*, 2018, 2127–36.

[btae236-B13] Ghaffari Laleh N , Muti HS, Loeffler CML et al Benchmarking weakly-supervised deep learning pipelines for whole slide classification in computational pathology. Med Image Anal 2022;79:102474. 35588568 10.1016/j.media.2022.102474

[btae236-B14] Lazard T , Bataillon G, Naylor P et al Deep learning identifies morphological patterns of homologous recombination deficiency in luminal breast cancers from whole slide images. Cell Rep Med 2022;3:100872. 36516847 10.1016/j.xcrm.2022.100872 PMC9798078

[btae236-B15] Lu MY , Williamson DF, Chen TY et al Data-efficient and weakly supervised computational pathology on whole-slide images. Nat Biomed Eng 2021;5:555–70. 33649564 10.1038/s41551-020-00682-w PMC8711640

[btae236-B16] Lu W , Toss M, Dawood M et al Slidegraph+: whole slide image level graphs to predict Her2 status in breast cancer. Med Image Anal 2022;80:102486. 35640384 10.1016/j.media.2022.102486

[btae236-B17] Naik N , Madani A, Esteva A et al Deep learning-enabled breast cancer hormonal receptor status determination from base-level H&E stains. Nat Commun 2020;11:5727. 33199723 10.1038/s41467-020-19334-3 PMC7670411

[btae236-B18] Pisula JI , Datta RR, Valdez LB et al Predicting the Her2 status in oesophageal cancer from tissue microarrays using convolutional neural networks. Br J Cancer 2023;128:1369–76. 36717673 10.1038/s41416-023-02143-y PMC10050393

[btae236-B19] Seol H , Lee HJ, Choi Y et al Intratumoral heterogeneity of Her2 gene amplification in breast cancer: its clinicopathological significance. Mod Pathol 2012;25:938–48. 22388760 10.1038/modpathol.2012.36

[btae236-B20] Song AH , Jaume G, Williamson DF et al Artificial intelligence for digital and computational pathology. Nat Rev Bioeng 2023;1:930–49.

[btae236-B21] Swain SM , Shastry M, Hamilton E. Targeting Her2-positive breast cancer: advances and future directions. Nat Rev Drug Discov 2023;22:101–26. 36344672 10.1038/s41573-022-00579-0 PMC9640784

[btae236-B22] Tsai P-C , Lee T-H, Kuo K-C et al Histopathology images predict multi-omics aberrations and prognoses in colorectal cancer patients. Nat Commun 2023;14:2102. 37055393 10.1038/s41467-023-37179-4 PMC10102208

[btae236-B23] Vaswani A , Shazeer N, Parmar N et al Attention is all you need. Adv Neural Inform Process Syst 2017;30:5998–6008.

[btae236-B24] Vu QD , Rajpoot K, Raza SEA et al Handcrafted histological transformer (H2T): unsupervised representation of whole slide images. Med Image Anal 2023;85:102743. 36702037 10.1016/j.media.2023.102743

[btae236-B25] Wagner SJ , Reisenbüchler D, West NP, et al Transformer-based biomarker prediction from colorectal cancer histology: a large-scale multicentric study. Cancer Cell 2023;41:1650–61.e4. 37652006 10.1016/j.ccell.2023.08.002 PMC10507381

[btae236-B26] Wang X , Du Y, Yang S et al RetCCL: clustering-guided contrastive learning for whole-slide image retrieval. Med Image Anal 2023;83:102645. 36270093 10.1016/j.media.2022.102645

[btae236-B27] Wang Y , Hu C, Kwok T et al Demos: a deep learning-based ensemble approach for predicting the molecular subtypes of gastric adenocarcinomas from histopathological images. Bioinformatics 2022;38:4206–13. 35801909 10.1093/bioinformatics/btac456

[btae236-B28] Wolff AC , Hammond MEH, Allison KH et al Human epidermal growth factor receptor 2 testing in breast cancer: American society of clinical oncology/college of American pathologists clinical practice guideline focused update. Arch Pathol Lab Med 2018;142:1364–82. 29846104 10.5858/arpa.2018-0902-SA

[btae236-B29] Yu J-G , Wu Z, Ming Y et al Prototypical multiple instance learning for predicting lymph node metastasis of breast cancer from whole-slide pathological images. Med Image Anal 2023;85:102748. 36731274 10.1016/j.media.2023.102748

