# Supplementary material for: PhiHER2: phenotype-informed weakly supervised model for HER2 status prediction from pathological images
Source: Bioinformatics. 2024 Jun 28;40(Suppl 1):i79–90. doi: 10.1093/bioinformatics/btae236 (PMC11211833; doi:10.1093/bioinformatics/btae236)
Supplement: btae236_Supplementary_Data [file btae236_supplementary_data.pdf]

Supplementary Materials

PhiHER2: Phenotype-informed weakly supervised model for HER2 status prediction from pathological images

Supplementary Notes

S1. Implementation details

Our PhiHER2 model was implemented using Python 3.8 and the PyTorch 2.0 deep learning framework. All models, including comparative methods, were conducted on one workstation with a 12GB NVIDIA GPU. The CUDA version is 12.1 and the GPU driver version is 530.41. Adam algorithm was employed for parameters optimization. The momentum factor was 0.9, and the learning rate was initially set to 0.0001. LinearLR scheduler aimed to the learning rate decays was operated and set to a limit with 0.00001 when the number reaches epoch 500. The maximum number of epochs was set to 1000, and an early stopping strategy was utilized. It is determined by monitoring the loss value unchanged on the validation set with patience 50. A gradient accumulation strategy was also adopted to address the issue of an inconsistent number of patches across WSIs. The Gradient accumulation size was set to 32, which is equivalent to using a minibatch size of 32. The weights of all layers were initialized with the Kaiming uniform strategy. The optimized model with the lowest loss metric value on the validation set was recorded and utilized for the following evaluation on the test set. WSIs were processed by OpenSlide Toolkit v3.4.1. For hierarchical prototype clustering, validation and test WSIs were excluded to prevent data leakage.

S2. Evaluation metrics

The performance of our approach and comparative methods were evaluated via multiple metrics, including the Receiver Operating Characteristic (ROC) curve with its Area Under the Curve (AUC) value, the Precision-Recall (PR) curve with the Area Under the PR Curve (AUPRC), the balanced Accuracy (bACC), and the F1 score for both the negative and positive classes (negF1 and posF1), as well as the weighted average of the Precision, Recall, and F1 score (denoted as wPRC, wREC, and wF1). In the definition of posF1, true positive is the number of positive cases classified as positive.

For the HEROHE dataset, which includes a training set and an independent test set, we randomly divided 20% of the training slides into a validation set for model selection. This process was repeated for 5 times to alleviate the impact of data split. As a result, we obtained 5 models, their performance was evaluated on the independent HEROHE test set. The average results over the 5-time models are reported. The data split strategy is illustrated in Supplementary Fig. S1(a).

For the Yale HER2 cohort, we employed a 5-time 5-fold cross validation scheme. In the experiment of each time, we randomly divided 20% of the samples for testing (marked as d1), while

the remaining samples were evenly divided into 5 non-overlapping subsets (folds). We then iteratively trained the model on 4 subsets (4 folds) while using the remaining subset for validation. This process was repeated for each fold, ensuring that each fold served as the validation subset exactly once. The trained models corresponding to the 5 folds were evaluated on the testing data (d1), and the results were averaged. This experiment was repeated for 5 times. The average results over the 5-time experiments are reported. This data splitting strategy is illustrated in Supplementary Fig. S1(b).

Due to the distinct data characteristics between the two datasets, we did not conduct a cross-study test (trained on one dataset and tested on the other) in our work.

S3. Comparative methods

To verify the effectiveness of our PhiHER2 model, we compared it with existing state-of-the-art methods in WSIs classification. Brief introductions of the comparative methods are summarized as follows:

- Attention-based multi instance learning (ABMIL) (Ilse, Tomczak, and Welling 2018), which is a baseline MIL method for current WSIs classification in the field of CPath.
- CLAM, a clustering-constrained-attention MIL method (Lu et al. 2021) for pathological subtyping. It relies on instance-level clustering to refine the feature space in MIL.
- Prototypical multiple instance learning (PMIL) (Yu et al. 2023). It introduces prototypes into vocabulary-based MIL for lymph node metastasis prediction on WSIs.
- A transformer-based architecture which was applied for end-to-end biomarker prediction from WSIs (Wagner et al. 2023). It is denoted as TRANS here. We halved the architecture to a smaller scale with 4 attention heads due to data limitation in our experiments.

References

Ilse, Maximilian, Jakub Tomczak, and Max Welling. 2018. “Attention-Based Deep Multiple Instance Learning.” In *Proceedings of the 35th International Conference on Machine Learning*, 2127–36. PMLR. https://proceedings.mlr.press/v80/ilse18a.html.

Lu, Ming Y., Drew F. K. Williamson, Tiffany Y. Chen, Richard J. Chen, Matteo Barbieri, and Faisal Mahmood. 2021. “Data-Efficient and Weakly Supervised Computational Pathology on Whole-Slide Images.” *Nature Biomedical Engineering* 5 (6): 555–70. https://doi.org/10.1038/s41551-020-00682-w.

Wagner, Sophia J., Daniel Reisenbüchler, Nicholas P. West, Jan Moritz Niehues, Jiefu Zhu, Sebastian Foersch, Gregory Patrick Veldhuizen, et al. 2023. “Transformer-Based Biomarker Prediction from Colorectal Cancer Histology: A Large-Scale Multicentric Study.” *Cancer Cell* 41 (9): 1650-1661.e4. https://doi.org/10.1016/j.ccell.2023.08.002.

Yu, Jin-Gang, Zihao Wu, Yu Ming, Shule Deng, Yuanqing Li, Caifeng Ou, Chunjiang He, Baiye Wang, Pusheng Zhang, and Yu Wang. 2023. “Prototypical Multiple Instance Learning for Predicting Lymph Node Metastasis of Breast Cancer from Whole-Slide Pathological Images.” *Medical Image Analysis* 85 (April): 102748. https://doi.org/10.1016/j.media.2023.102748.

Supplementary Tables

Table S1. Comparison of inference time cost for our PhiHER2 and other comparative methods. An ImageNet-based pre-trained Resnet18 (a full-supervised learning model) was also employed for inference time estimation. The inference process of our PhiHER2 and all comparative methods were conducted on the same hardware setup (Ubuntu 20.04.5 LTS, Intel(R) Core(TM) i7-9700 CPU @ 3.00GHz, NVIDIA GeForce RTX 3060 with 12GB graphics memory (VRAM), and 32GB physical memory size (RAM)). The total times taken to infer all test data are reported. The best results are marked in **bold**.

Inference time (sec.)	**Full-supervised learning models**	**Weakly-supervised learning models**						
	Resnet18 (patch-level)	PhiHER2	PMIL-Cosine	PMIL-Euclidean	CLAM-B8	CLAM-B32	ABMIL	TRANS
HEROHE	856	43.45	40.00	**39.75**	45.00	45.21	46.41	56.62
Yale	281	19.61	15.15	**14.82**	30.42	31.50	32.84	35.51

Table S2. Comparison results on our PhiHER2 model against top entries of the public leaderboard. The results were evaluated on the HEROHE test dataset. The evaluation metrics are from the public leaderboard. The best results are marked in **bold**. "NA" denotes no results are reported in the public leaderboard.

Rank	Team	F1 Score	AUC	Recall	Precision	Accuracy
*	PhiHER2	**0.706±0.02**	**0.795±0.03**	**0.837±0.07**	**0.613±0.03**	**0.721±0.02**
Top 1	Macaroon	0.68	0.71	0.83	0.57	NA
Top 2	MITEL	0.67	0.74	0.78	0.58	NA

Table S3. Ablation results for cluster phenotype guidance with the cross-attention module evaluated on the HEROHE and Yale dataset. The baseline model corresponds to the ABMIL architecture, while the Cluster-PT is equivalent to our PhiHER2 approach. The best results are indicated in **bold**.

Dataset	Config.	wPRC	wREC	wF1	bACC	AUC
HEROHE	Baseline	0.706	0.655	0.655	0.680	0.724
	Non-PT	0.655	0.680	0.651	0.670	0.724
	Rand-PT	0.673	0.652	0.638	0.656	0.694
	Initial-PT	0.751	0.699	0.696	0.719	0.785
	**Cluster-PT**	**0.762**	**0.721**	**0.722**	**0.741**	**0.795**
Yale	Baseline	0.775	0.767	0.766	0.767	0.820
	Non-PT	0.770	0.765	0.764	0.765	0.826
	Rand-PT	0.687	0.680	0.664	0.680	0.764
	Initial-PT	0.821	0.815	0.814	0.815	0.892
	**Cluster-PT**	**0.829**	**0.819**	**0.817**	**0.819**	**0.893**

Table S4. Comparison results for classifier head strategies evaluated on the HEROHE and Yale dataset. The baseline model corresponds to the ABMIL architecture. The best results are indicated in **bold**.

Dataset	Cls. head	wPRC	wREC	wF1	bACC	AUC
HEROHE	Baseline	0.706	0.655	0.655	0.680	0.724
	Transformer	0.731	0.696	0.695	0.708	0.768
	Attention score	0.694	0.671	0.674	0.680	0.739
	**Mean**	**0.762**	**0.721**	**0.722**	**0.741**	**0.795**
Yale	Baseline	0.775	0.767	0.766	0.767	0.820
	Transformer	0.772	0.762	0.760	0.762	0.826
	Attention score	0.810	0.800	0.798	0.800	0.877
	**Mean**	**0.829**	**0.819**	**0.817**	**0.819**	**0.893**

Supplementary Figures

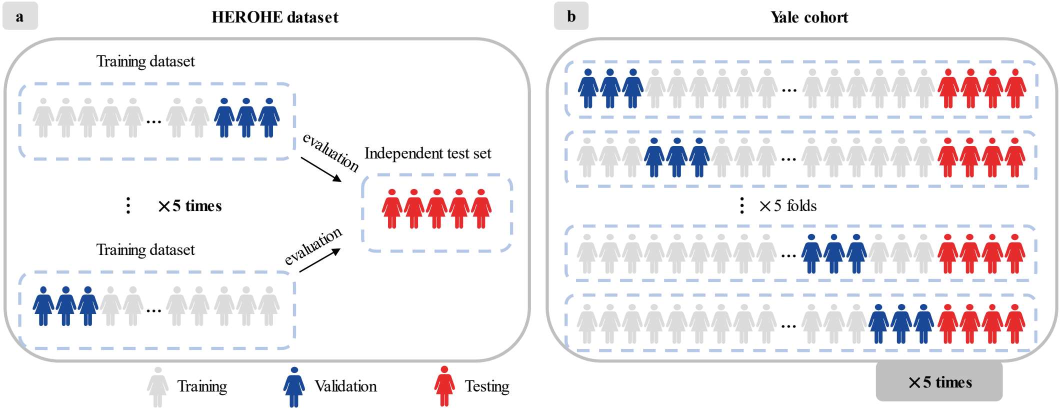

Figure S1. Illustration of the data splitting strategy with regard to training, validation, and testing for (a) the HEROHE dataset and (b) the Yale cohort.

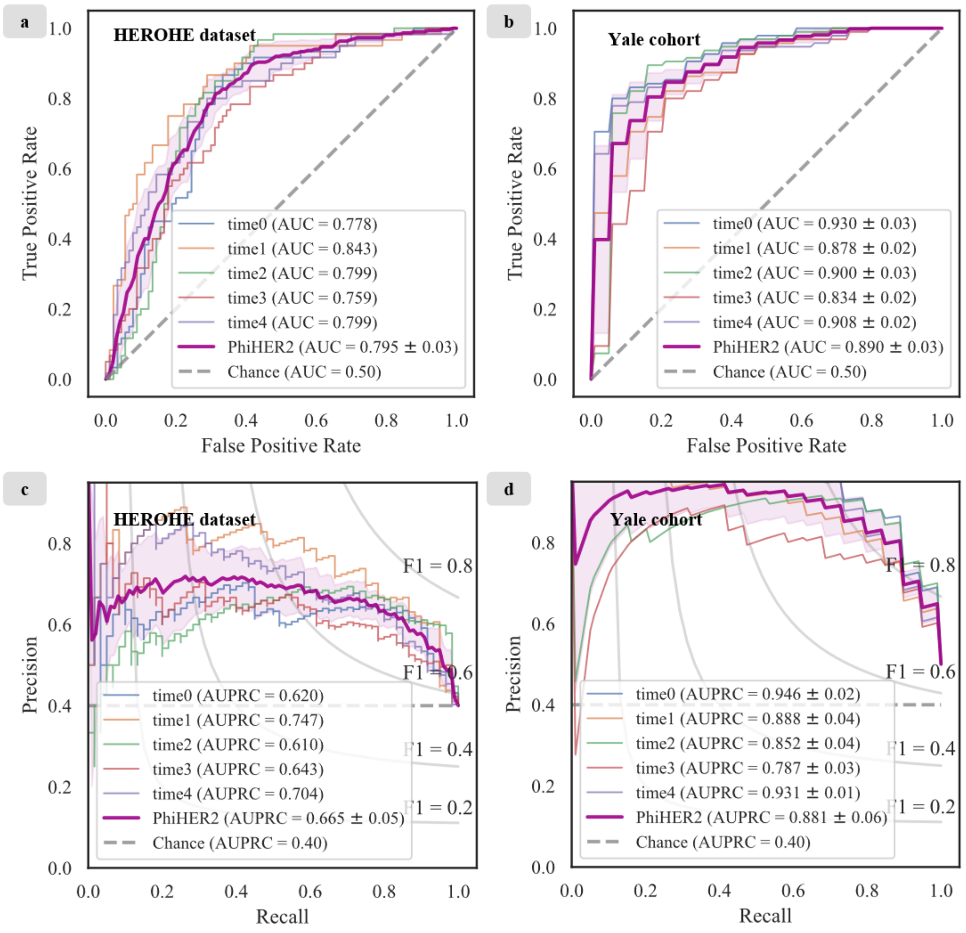

Figure S2. 5-time evaluation performance of our PhiHER2 model for HER2 status prediction with (a-b) ROCs on the HEROHE and Yale cohort, and (c-d) PR curves.

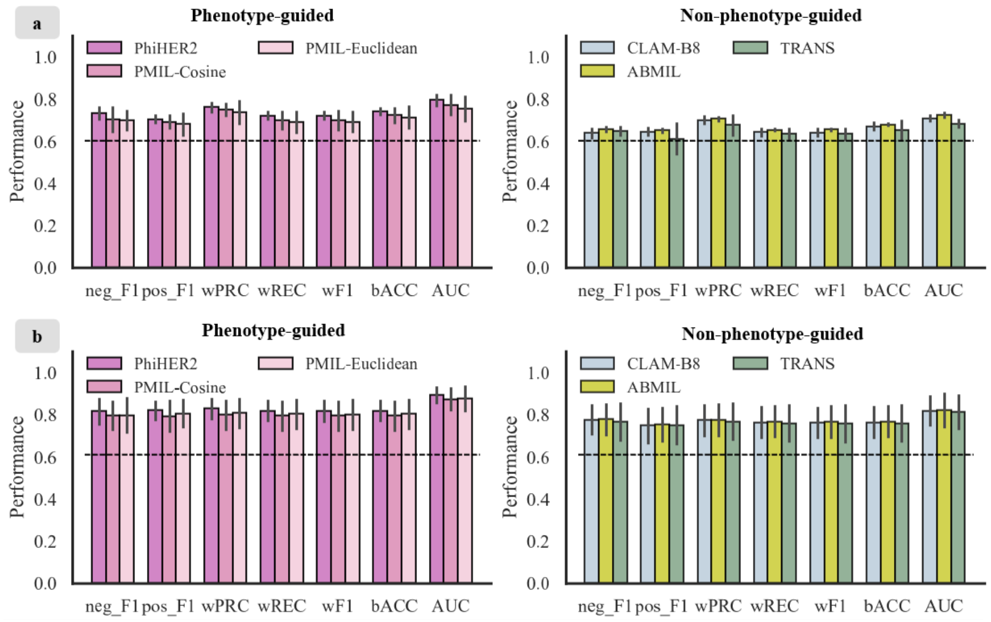

Figure S3. Evaluation performance of all comparative methods about phenotype-guided and non-phenotype-guided groups on (a) the HEROHE dataset and (b) the Yale cohort.

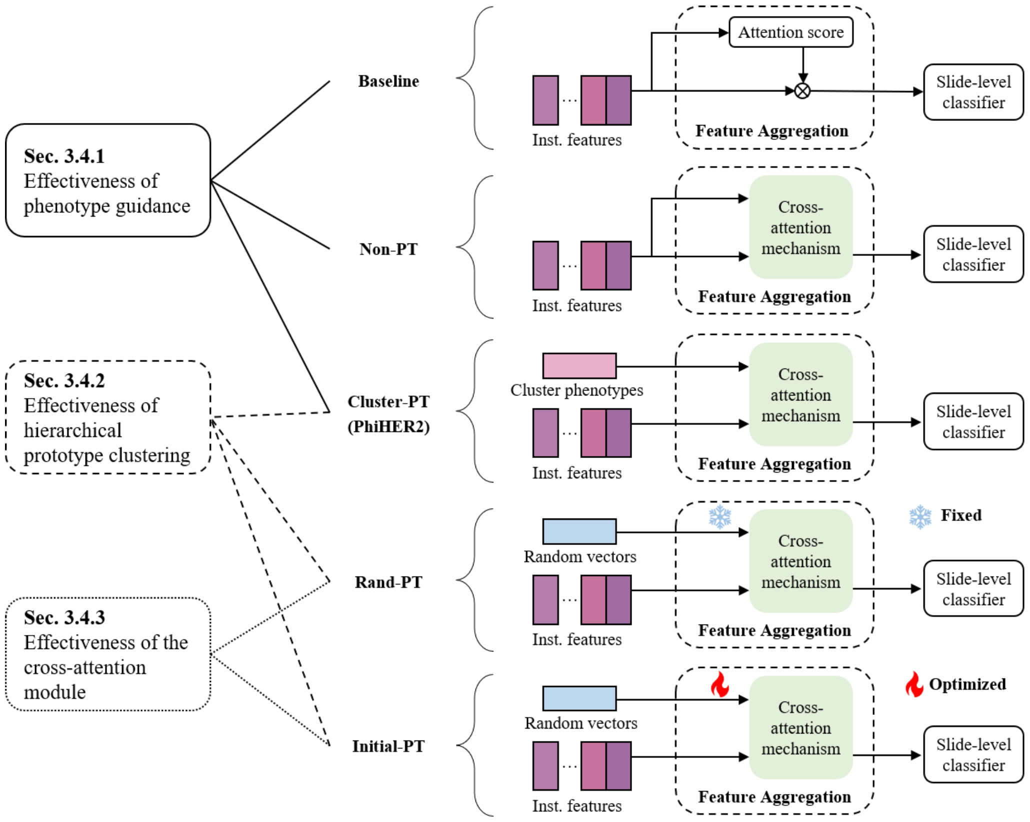

Figure S4. Illustration of different experimental configurations, including Baseline, Non-PT, Cluster-PT, Rand-PT, and Initial-PT models. The comparative models in their respective subsections are combined and connected by lines for clarity (Solid line: Sec. 3.4.1, Short dashed line: Sec. 3.4.2, and dotted dashed line: Sec. 3.4.3).

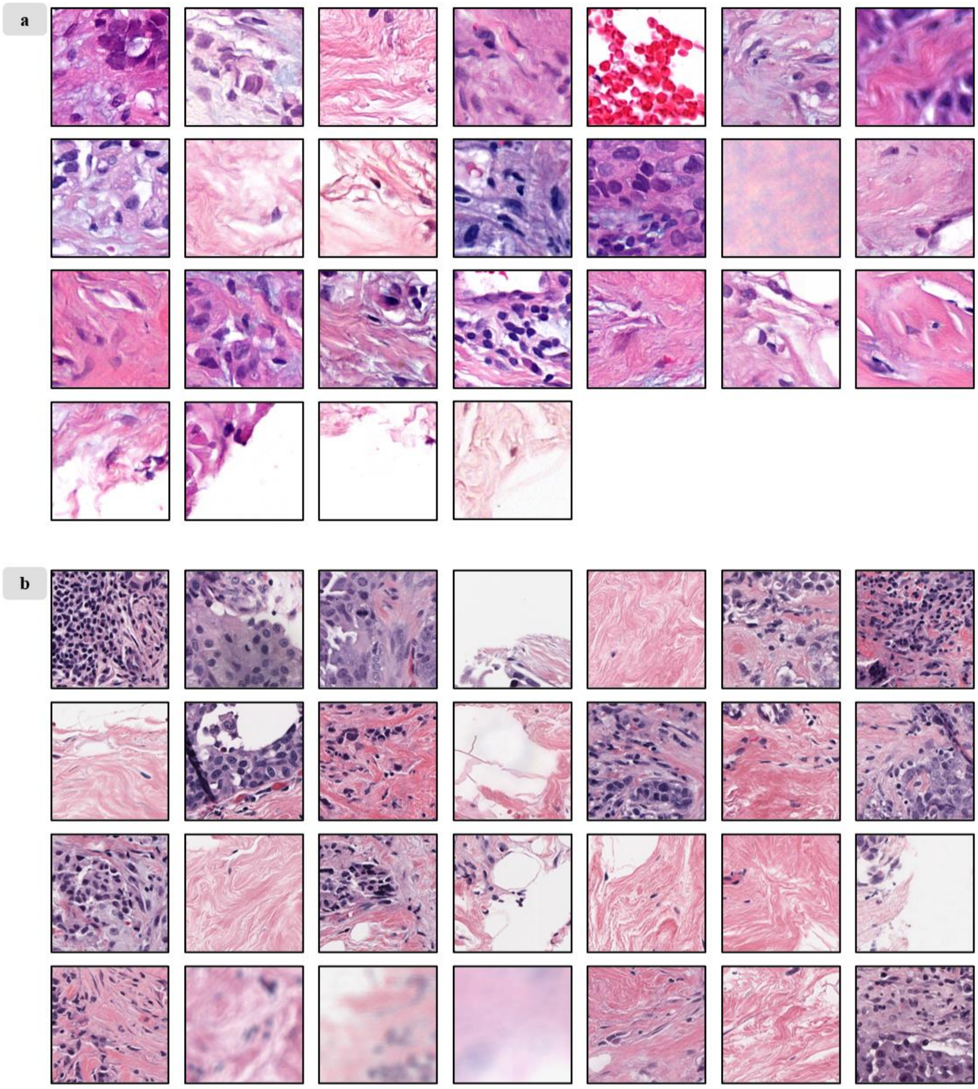

Figure S5. Visualization of entire phenotype patches derived from the hierarchical prototype clustering module for time-0 model on (a) the HEROHE dataset and (b) the Yale cohort.

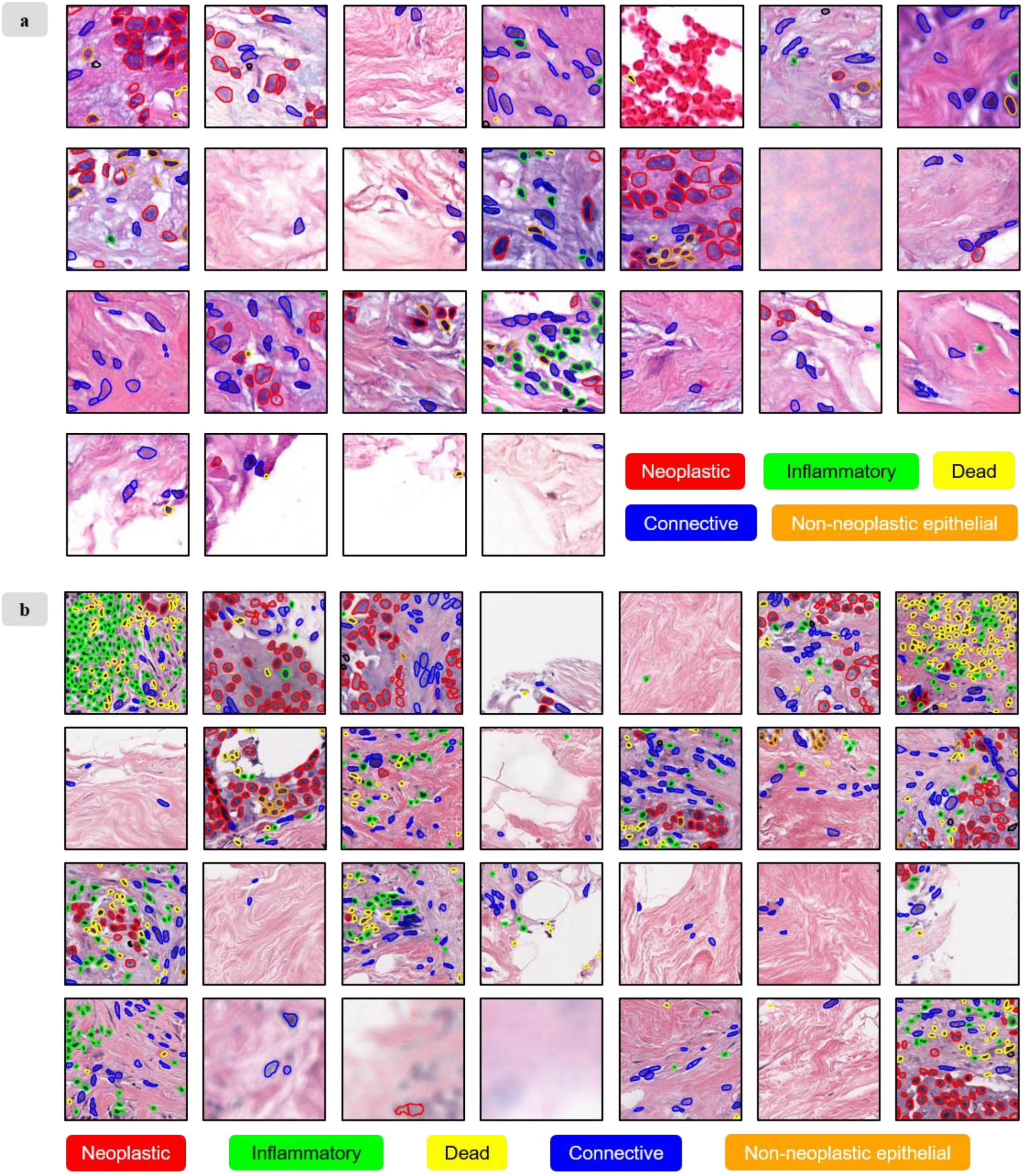

Figure S6. Visualization of cell segmentation and classification results overlaid on phenotype patches from (a) the HEROHE dataset and (b) the Yale cohort. These phenotype patches are derived from the hierarchical prototype clustering module in time-0 model. Different colors marked different cell types.

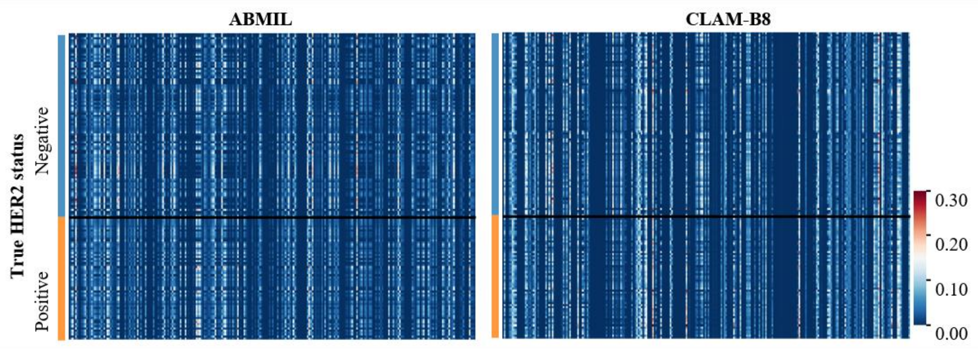

Figure S7. Visualization of WSI-level representations on the HEROHE test cases for ABMIL and

CLAM methods.

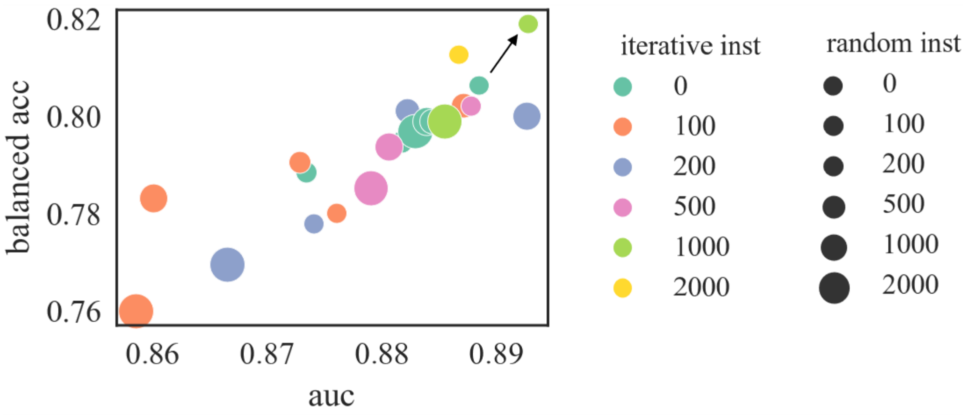

Figure S8. Evaluation performance in terms of balanced accuracy and AUC for dual instance sampling on the Yale cohort. Each point signifies a distinct model, where the size corresponds to the value of random sampling, and the number of instances in iterative mining is denoted by various colors.

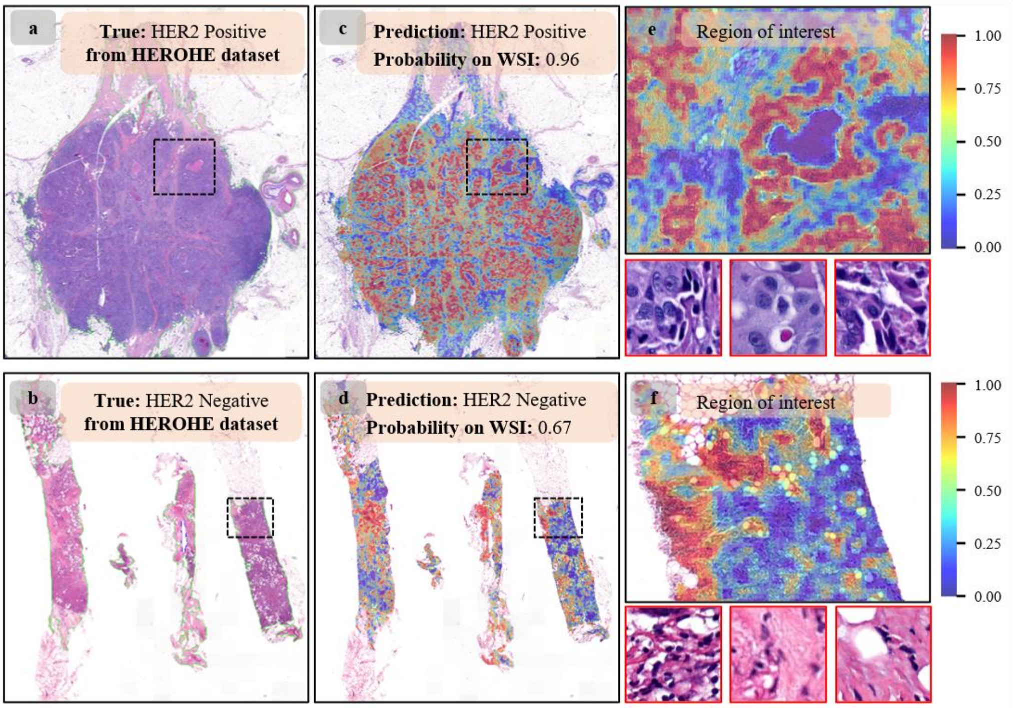

Figure S9. (a-b) Pathological WSIs from the HEROHE dataset with automated ROIs identification. (c-d) The corresponding attention heatmaps overlapped on raw WSIs. Large values (red) means a high contribution to the model prediction, and small values (blue) a low contribution. (e-f) Selected ROIs for zooming in to observe detailed tissue regions. 3 tissue patches with the highest probabilities are also presented.
